# Supplementary material for: Magnetic Nanoparticle Support with an Ultra-Thin Chitosan Layer Preserves the Catalytic Activity of the Immobilized Glucose Oxidase
Source: Nanomaterials (Basel). 2024 Apr 17;14(8):700. doi: 10.3390/nano14080700 (PMC11054521; doi:10.3390/nano14080700)
Supplement: Supplementary file 1 [file nanomaterials-14-00700-s001.zip › nanomaterials-2961466-supplementary.pdf]

Supplementary materials

Materials

Iron (III) chloride hexahydrate (99%) ($FeCl_3 \cdot 6\ H_2O$), iron (II) chloride tetrahydrate (99%) ($FeCl_2 \cdot 4\ H_2O$), D-glucose (99.5%), ammonium hydroxide solution (25%), phosphate buffered saline (PBS buffer), acetic acid, and sodium tripolyphosphate were purchased from Nevareaktiv (Russia) and used without purification. Glucose oxidase (GOx) lyophilized powder (*Aspergillus niger,* 174.9 U/mg), peroxidase (HPR, 150 U/mg), and glutaraldehyde solution (GA, 25%) were purchased from Sigma-Aldrich and used as received. Chitosan middle-viscous, insoluble matter <1%, 400KDa and N-(3-dimethylamiopropyl)-N'-ethyl carbodiimide hydrochloride (99%) were obtained from FLUKA, BioChemika (Japan). N-hydroxysuccinimide (98%) was purchased from Acros Organics (China). ABTS (2,2′-Azino-bis(3-ethylbenzothiazoline-6-sulfonic acid) diammonium salt, 98%) was obtained from AppliChem, Panreac, and used as received. Ethanol was purchased from Pharmco-Aaper and used without purification,

Synthesis of MNA

In a typical experiment, 15 mL of the $FeCl_3{\times}6H_2O$ solution (2.162 g in 15 mL of distilled water) and 15 mL of the $FeCl_2{\times}4H_2O$ solution (0.792 g in 15 mL of distilled water) were mixed in a jacketed glass reactor. The mixture was stirred for 15 min at a rate of 400 min $^{-1}$ at room temperature, after which the temperature in the reactor was increased to 65 °C. The mixture was stirred for another 15 min. After that, 10 mL of aqueous ammonia solution (25%) was added dropwise at a rate of 2 mL /min followed by stirring for another 15 min.

Biocatalyst activity assays

In a typical experiment, 1.5 mL of D-glucose (50 mM), 1.5 mL of ABTS (0.02 M), and 15 µL of native GOx (0.05 g) solution or 0.25g of the biocatalyst containing 0.05 g of GOx were mixed with 1.8 mL of the PBS buffer at pH 6.0, followed by the addition of 200 µL of HRP (10 µg.mL^{-1}). Then the solutions were incubated for five minutes, after which the absorbance was measured using a UV-5 spectrophotometer (UV/VIS Mettler Toledo) at 415 nm. At small D-glucose concentrations, the amount of the reacted substrate was additionally determined by iodometric titration [1]. It is noteworthy that in the enzyme activity measurements both native and immobilized GOx samples had the same concentration of GOx and each experiment was repeated three times.

The biocatalyst activity (A, mM(Gl_r)/(mg (GOx) ×min)) was calculated using Eq. (S1)

[2,3]:

$$A = \frac{Gl_r}{[GOx]\times IC\times V\times \tau} \qquad \text{(S1)}$$

where $[GOx]$ is the amount (mg) of native or immobilized GOx, V is the volume of the reaction solution, τ is the reaction time, and Gl_r is the amount of D-glucose (mmol) reacted. The immobilization coefficient (IC) [4] was calculated as a change of the GOx activity in the supernatant before and after immobilization of GOx on the support in the enzyme solution.

The maximum consumption of the substrate was regarded 100%. Then the relative activity (R) was calculated as follows (Eq. S2) [2,5,6]:

Relative activity (%) = enzymatic activity/maximum enzymatic activity×100% (S2).

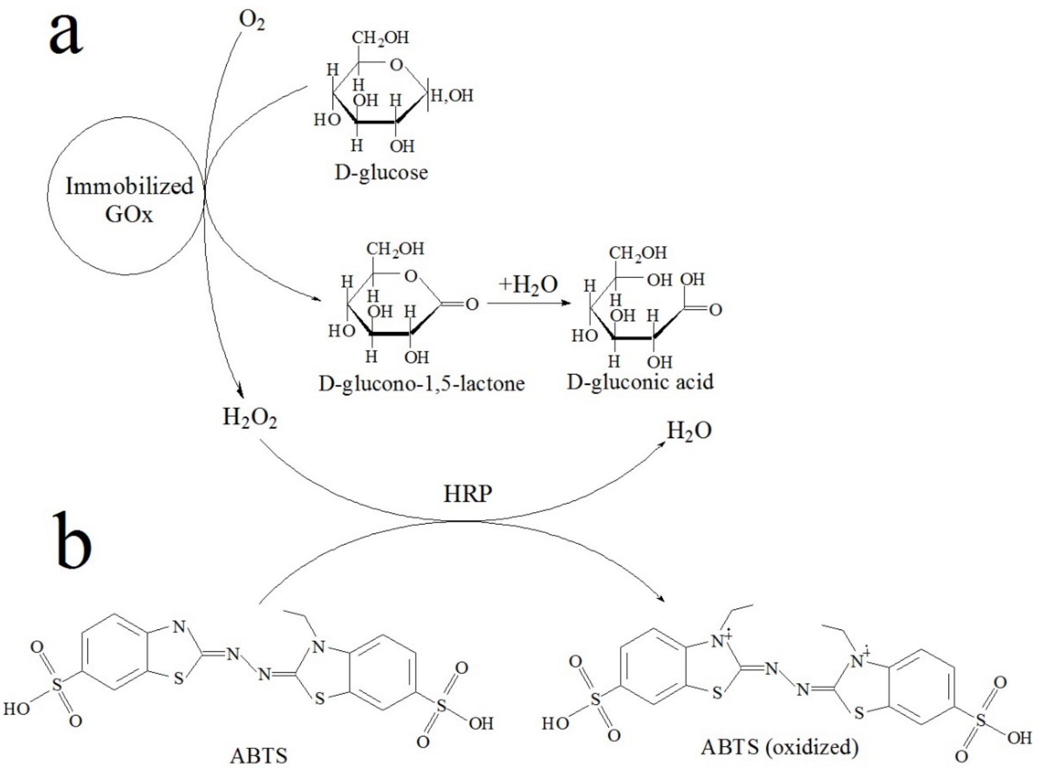

Scheme S1. Schematic representation of D-glucose oxidation to D-gluconic acid in the presence of HRP and ABTS.

Reusability of biocatalysts

In a typical experiment for the biocatalyst reuse, the biocatalyst was separated with a rare earth magnet after each catalytic reaction and triple washed using a PBS buffer at pH 6 in order to remove the remaining substrate that could be attached to the support surface. Finally, the biocatalyst was added to the next reaction mixture.

Characterization

Nitrogen adsorption measurements were carried out at liquid nitrogen temperature on an Beckman Coulter TM SA 3100TM (Coulter Corporation, USA) using a sample preparation device Beckman Coulter TM SA-PREPTM (Coulter Corporation, USA). Samples were degassed at 100 °C in vacuum. The total surface area was estimated by the *Brunauer–Emmett–Teller* (BET) method, while the pore size distribution was determined by the *Barrett–Joyner–Halenda* (BJH) method using desorption.

Bright-field scanning transmission electron microscopy (BF STEM) and high resolution (HRTEM) images as well as energy dispersive spectroscopy (EDS) were acquired on Osiris at 200 kV. The samples were prepared by placing a drop of the material suspension on the carbon coated TEM grid.

Magnetization measurements of the samples were carried out in a home-built vibrating sample magnetometer (VSM) with accuracy better than ±0.01 emu/g, which allows measuring bulk and powder samples of 0.01-150 mg in temperature range 80-1000 K and magnetic field 0-2.5 T.

Thermal gravimetric analysis (TGA) was performed on TG IRIS209 F1 NETZSCH placing ~5-10 mg of the powder in aluminum pans. The experiments were carried out upon heating to 600 ºC at a rate of 10.0 ºC/min.

X-ray photoelectron spectroscopy (XPS) data were obtained using MgK_ (h_ = 1253.6 eV) radiation with the ES-2403 spectrometer (Institute for Analytical Instrumentation of the RAS, St.Petersburg, Russia) equipped with an energy analyzer PHOIBOS 100-MCD5 (SPECS, Berlin, Germany) and a X-ray source XR-50 (SPECS, Berlin, Germany). All the data were acquired at the X-ray power of 250W. Survey spectra were recorded at an energy step of 0.5 eV with an analyzer pass energy of 40 eV. Samples were allowed to outgas for 180 min before analysis and were stable during the examination. The data analysis was performed by CasaXPS.

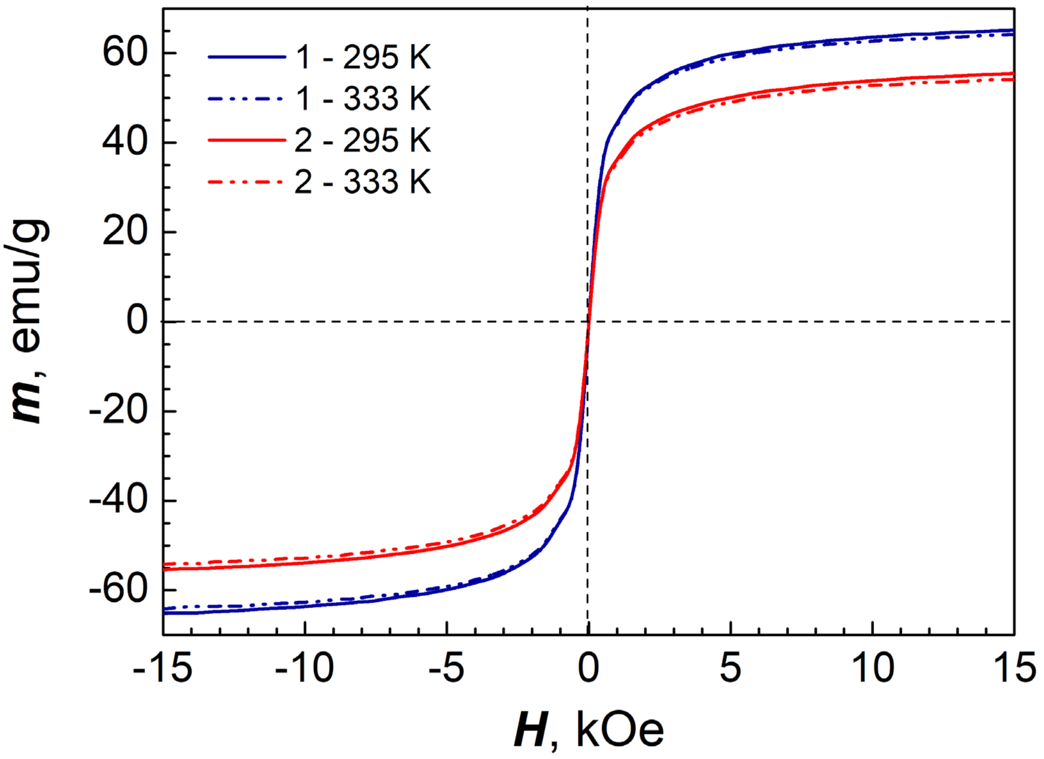

Figure S1. Hysteresis curves of MNA (1) and MNA-CSP (2).

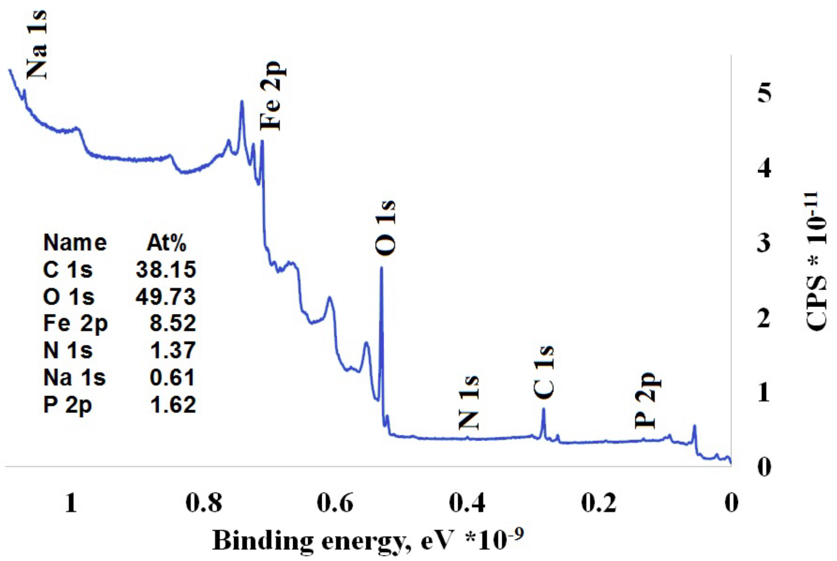

Figure S2. XPS survey spectrum of MNA-CSP.

Table S1. BET surface areas, pore volumes, and pores sizes for MNA, MNA-CSP, and MNA-CSP-GOx.

Sample notation	BET surface area, m^2/g	BET pore volume, cm^3/g	Mean pores size, nm
MNA	98	0.32	16
MNA-CSP	91	0.28	12
MNA-CSP-GOx	90	0.27	12

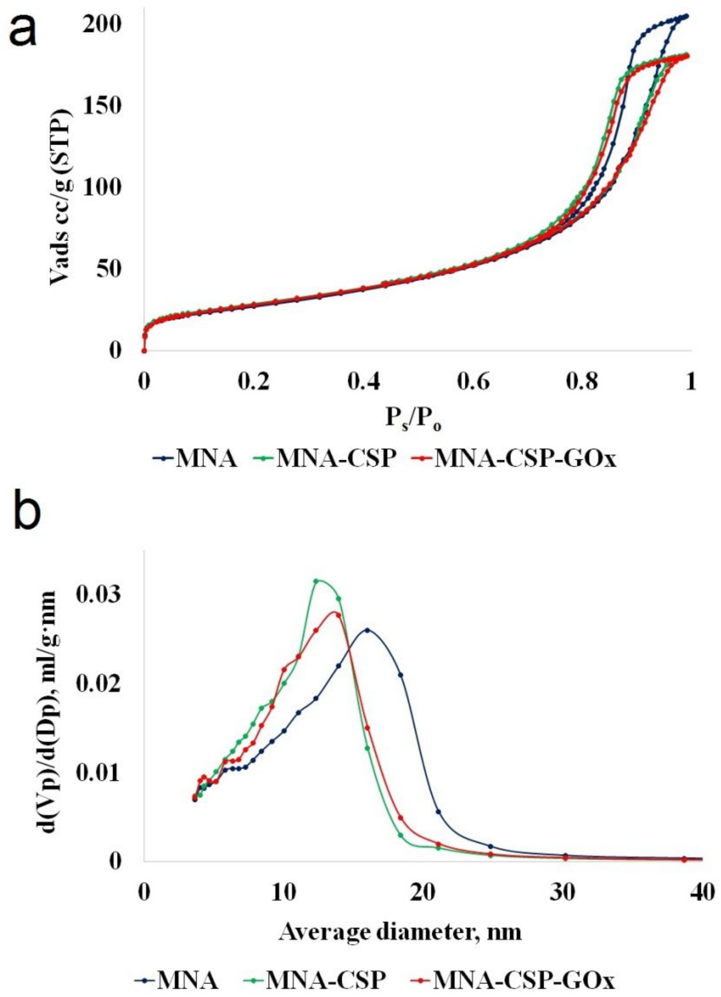

Figure S3. Liquid nitrogen adsorption-desorption isotherms (a) and pore size distributions (b) for MNA, MNA-CSP, and MNA-CSP-GOx.

Kinetic measurements

To determine kinetics, the initial reaction rates for both native and immobilized GOx, were obtained at varied concentrations of D-glucose (2.2-22 mM) in the PBS buffer at pH 6.0 and 35 °C. The data obtained are presented in Figure S4.

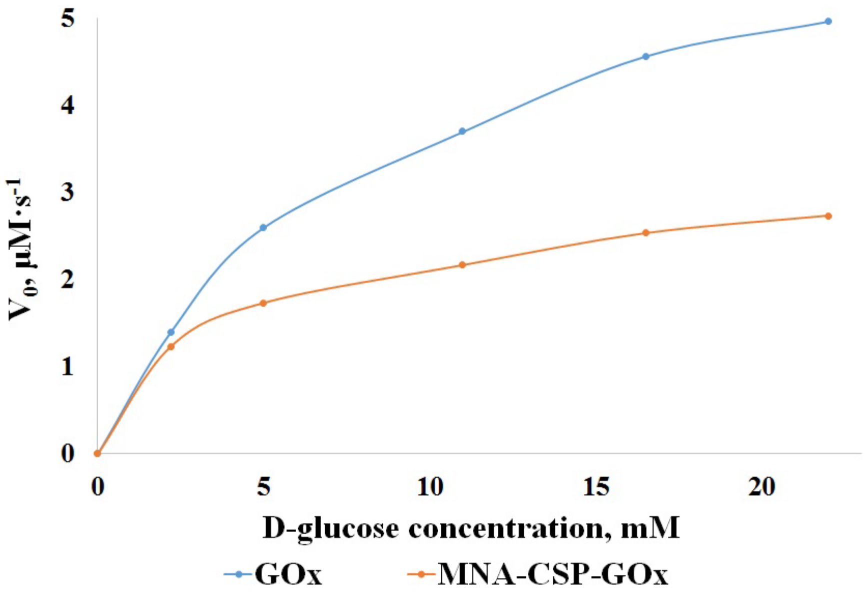

Figure S4. The dependence of the initial reaction rates on D-glucose concentrations.

For an enzymatic reaction, the dependence of the reaction rate (V) on the substrate concentration ([S]) was obtained from the Michaelis-Menten equation, whose parameters can be obtained from the Lineweaver-Burk equation (S1):

$$\frac{1}{V} = \frac{K_m}{V_{max}} \times \frac{1}{[S]} + \frac{1}{V_{max}} \quad \text{(S1)}$$

The Lineweaver-Burk graphs (the dependence of 1/V on 1/[S]) were obtained for native and immobilized GOx as is shown in Figure S5. The straight line obtained crossing the Y axis gives 1/V max, while crossing the X axis provides $-1/K_m$. The tilt angle is K_m/V_{max},

where V is the initial reaction rate, V_{max} is the maximum reaction rate, K_m is the Michaelis constant, and [S] is the initial substrate concentration.

Based on these data, the maximum reaction rate (V_{max}) and the Michaelis-Menten constant (K_m) were computed using the Lineweaver-Burk analysis [6,7].

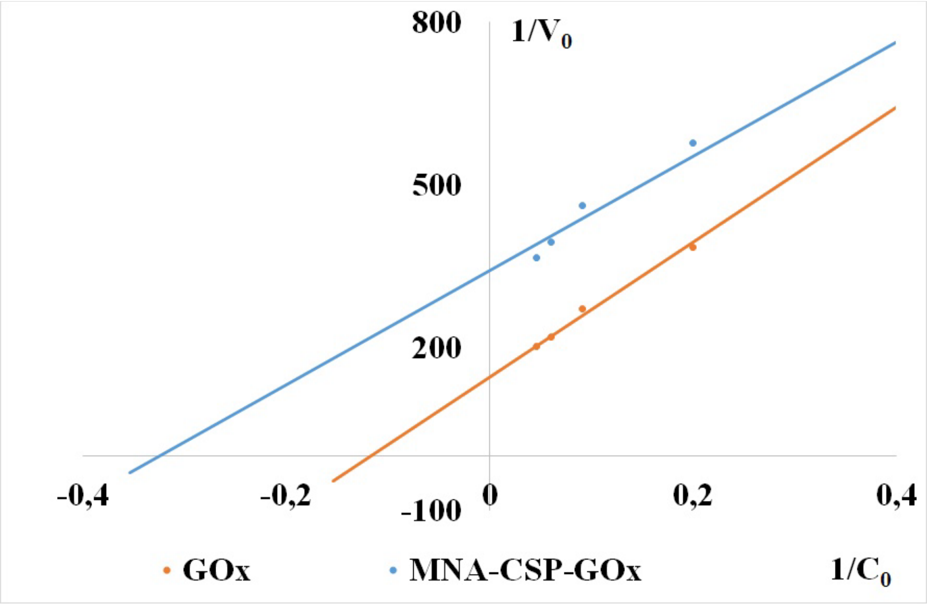

Figure S5. Lineweaver-Burk graphs of free GOx and MNC-CSP-GOx.

Table S2. Relative activity of MNA-CSP-GOx in recycling.

Cycle number	1	2	3	4	5	6	7	8	9	10
Relative activity, %	100	98.0	94.8	93.5	91.1	88.0	87.4	84.6	81.7	79.6

References

1. Graf, E.; Penniston, J.T. Method for determination of hydrogen peroxide, with its aaplication illustrated by glucose assay. *Clinical Chemistry* **1980**, *26*, 658-660.
2. Haskell, A.K.; Sulman, A.M.; Golikova, E.P.; Stein, B.D.; Pink, M.; Morgan, D.G.; Lakina, N.V.; Karpenkov, A.Y.; Tkachenko, O.P.; Sulman, E.M.; et al. Glucose Oxidase Immobilized on Magnetic Zirconia: Controlling Catalytic Performance and Stability. *ACS Omega* **2020**, *5*, 12329-12338, doi:10.1021/acsomega.0c01067.
3. Acosta, L.C.; Perez Goncalves, G.M.; Pielak, G.J.; Gorensek-Benitez, A.H. Large cosolutes, small cosolutes, and dihydrofolate reductase activity. *Protein Sci.* **2017**, *26*, 2417-2425, doi:10.1002/pro.3316.
4. Nguyen, L.T.; Yang, K.-L. Combined cross-linked enzyme aggregates of horseradish peroxidase and glucose oxidase for catalyzing cascade chemical reactions. *Enzyme Microb. Technol.* **2017**, *100*, 52-59, doi:10.1016/j.enzmictec.2017.02.007.
5. Zhao, B.; Zhou, L.; Ma, L.; He, Y.; Gao, J.; Li, D.; Jiang, Y. Co-immobilization of glucose oxidase and catalase in silica inverse opals for glucose removal from commercial isomaltooligosaccharide. *Int. J. Biol. Macromol.* **2018**, *107*, 2034-2043, doi:10.1016/j.ijbiomac.2017.10.074.
6. Liao, L.; Meng, Y.; Wang, R.; Jia, B.; Li, P.; Wang, R.; Li, P. Coupling and Regulation of Porous Carriers Using Plasma and Amination to Improve the Catalytic Performance of Glucose Oxidase and Catalase. *Front Bioeng Biotechnol* **2019**, *7*, 426.

7. Tavares, T.S.; Torres, J.A.; Silva, M.C.; Nogueira, F.G.E.; da Silva, A.C.; Ramalho, T.C. Soybean peroxidase immobilized on δ-FeOOH as new magnetically recyclable biocatalyst for removal of ferulic acid. *Bioprocess Biosyst. Eng.* **2018**, *41*, 97-106, doi:10.1007/s00449-017-1848-1.
